# Comparison of methylation patterns generated from genomic and cell-line derived DNA using the Illumina Infinium MethylationEPIC BeadChip array

**DOI:** 10.1186/s13104-019-4853-4

**Published:** 2019-12-21

**Authors:** L. J. Smyth, J. Kilner, A. P. Maxwell, A. J. McKnight

**Affiliations:** Molecular Epidemiology Research Group, Centre for Public Health, Queen’s University of Belfast, Belfast, UK

**Keywords:** Cell-line, Diabetes, DNA, Epigenetics, Genomic, Methylation, MethylationEPIC

## Abstract

**Objectives:**

Genomic DNA (gDNA) is the optimal source of DNA for methylation analysis. This study compared methylation patterns in gDNA derived from blood with cell-line derived DNA (clDNA) from the same individuals. The clDNA had been generated via an Epstein-Barr virus transformation of the participant’s lymphocytes. This analysis sought to determine whether clDNA has the potential to be utilised in lieu of finite/unavailable gDNA in methylation analyses using Illumina Infinium MethylationEPIC BeadChip arrays that assess 862,927 CpG sites.

**Results:**

DNA samples were divided into two groups with eight gDNA and eight matched clDNA samples compared in each group (n = 16 individuals with 32 samples in total). Methylation patterns for gDNA samples generated for both groups were compared to the clDNA equivalent samples using Partek^®^ Genomics Suite^®^ to assess whether the significantly different CpG sites were consistent between both groups. In total, 28,632 CpG sites with significantly different levels of methylation (p < ×10^−8^) were common to both groups while 828,072 CpG sites assessed by the MethylationEPIC array were not significantly different in either group. This indicates that there is potential for clDNA to be used as a replacement for finite gDNA samples when absolutely necessary in DNA methylation studies.

## Introduction

DNA methylation is a key epigenetic feature, defined as the covalent addition of a methyl group to the 5-carbon position of a cytosine nucleotide at cytosine-phosphate-guanine (CpG) sites. These CpG sites frequently cluster within CpG islands that are repetitive sequences often located near gene promoters. DNA methylation has been associated with several complex diseases including chronic kidney disease (CKD) and diabetes [1–9].

The current gold-standard method of assessing DNA methylation patterns is through whole-genome bisulphite sequencing (WGBS), a method which provides single-nucleotide resolution and whole-genome coverage of approximately 95% of all CpG sites. However, this method requires large quantities of input DNA [10] and is financially prohibitive for many large-scale research studies. Illumina’s Infinium methylation arrays provide a user-friendly, cost-effective alternative, which require a lower input concentration of DNA [11, 12]. The Infinium MethylationEPIC array contains the most modern available technology and provides coverage of 862,927 CpG sites [13].

The aim of this study was to compare blood-derived genomic DNA (gDNA) and DNA derived from Epstein–Barr virus (EBV) transformed cell-lines (clDNA) from the same participants using data generated using Illumina’s Infinium MethylationEPIC BeadChip array. This provided an opportunity to evaluate methylation data generated from the more readily available clDNA samples compared to gDNA samples.

## Main text

### Methods

#### Sample cohort

All participants were of White ancestry from the British Isles and provided written informed consent for research. Each participant was recruited as part of the All Ireland-Warren 3-Genetics of Kidneys in Diabetes (GoKinD) UK Collection. DNA was frozen in multiple aliquots having been extracted from whole blood using the salting out method and normalised following PicoGreen quantitation. EBV transformation of participants’ peripheral blood leukocytes was performed by the European Collection of Authenticated Cell Cultures (ECACC) to create clDNA [14].

This study was conducted on 16 participants (with both gDNA and clDNA available for analyses). Eight participants were individuals with ≥ 10 years duration of type 1 diabetes (T1D) who had also been diagnosed with diabetic kidney disease (T1DKD) defined as persistent macroalbuminuria (≥ 500 mg/24 h), estimated glomerular filtration rate (eGFR) < 60 mL/min/m^2^ and hypertension (blood pressure ≥ 135/85 mmHg). The remaining eight individuals had ≥ 15 years duration of T1D and no evidence of renal disease on repeat testing (eGFR > 60 mL/min/m^2^). Duration of diabetes differed by ≤ 2 years and age at diagnosis ≤ 5 years. Participants were divided into two groups of eight and the overall characteristics are included within Table 1. Both gDNA and clDNA samples were analysed for all included individuals. Each case and control gDNA sample was compared to the matched clDNA sample generated from blood taken from the same individual.

**Table 1:** Characteristics of the individuals present within the Infinium MethylationEPIC BeadChip array analysis

Characteristics	Group 1	Group 2
Number of individuals	8 (4 gDNA + clDNA with DKD; 4 gDNA + clDNA with T1D and no renal disease)	8 (4 gDNA + clDNA with DKD; 4 gDNA + clDNA with T1D and no renal disease)
Average age of T1D diagnosis	15 years	17 years
Average duration of diabetes	35 years	30 years
Females:Males	5:3	5:3
Average BMI	27.0	24.4
Average BP (mmHg)	132/79	134/80
Hypertension	4	4
Smoking status	7 Never 1 Ex	6 Never 1 Ex 1 Current
eGFR > 60 mL/min/m^2^	4	4
eGFR 59–15 mL/min/m^2^ [CKD stages 3–4]	4	3
eGFR < 15 mL/min/m^2^ [ESRD]	0	1

*BMI* body mass index, *BP* blood pressure, *CKD* chronic kidney disease, *eGFR* estimated glomerular filtration rate, *ESRD* end-stage renal disease, *mmHg* millimetres of mercury, *T1D* type 1 diabetes

#### Infinium MethylationEPIC BeadChip array

Blood-derived DNA for each individual, both gDNA and clDNA (800 ng), was bisulphite treated (BST) using the EZ DNA Methylation™ Kit (Zymo Research, USA) using the manufacturer’s instructions. All samples were analysed together by the same individual, in the same laboratory.

To assess the methylation status of the CpG sites, the Infinium MethylationEPIC BeadChip array was used following the manufacturer’s instructions. This array quantitatively targets 862,927 CpG sites across the genome. Cases and controls were randomly distributed across each array. This high throughput platform evaluated individual methylation levels (β values) for each CpG site, ranging from 0 for unmethylated to 1 for complete methylation.

#### Quality control and statistical analyses

Raw methylation data was assessed for dye bias and quantile normalised as previously reported [1]. Quality control (QC) included evaluation of the bisulphite treatment conversion efficiency, dye specificity, hybridisation, and staining. This was assessed using GenomeStudio v2011 and BeadArray Controls Reporter software platforms (both Illumina).

MethylationEPIC analysis was performed using Partek^®^ Genomics Suite^®^ v7.19.1018. Only significant methylation values (p ≤ ×10^−8^) alongside a fold change of ± 2, generated in the analysis between gDNA and clDNA sample groups were included in the comparative analysis. Partek^®^ Genomics Suite^®^ was employed to complete Gene Ontology (GO) analysis and pathway enrichment analysis using the Kyoto Encyclopedia of Genes and Genomes (KEGG) database.

A schematic view of the methods undertaken in this manuscript is provided in Additional file 1: Appendix S1.

### Results

This research note is focused on the comparison of Infinium MethylationEPIC results for gDNA and clDNA samples, to determine whether clDNA has potential to be used in methylation array studies, in place of finite samples of gDNA. Each resulting.idat file generated from the iScan was assessed using Illumina’s BeadArray Controls Reporter software. This software assessed the data in connection with a pre-set standard set of controls. These QC results are included in Additional file 2: Table S1.

The resulting.idat files were analysed using Genome Studio v2011 and Partek^®^ Genomics Suite^®^ v7.19.1018. The total number of CpG sites examined by the Infinium MethylationEPIC array was 862,927. No significant difference in intensity levels was detected.

#### Differential methylation analysis between matched gDNA and clDNA samples for group 1 and group 2

Initially, the methylation patterns identified in the gDNA samples were directly compared to the clDNA equivalent samples within sample group 1 and then independently within sample group 2. Differentially methylated sites within each group were compared to assess whether the significantly different CpG sites were consistent between the two groups (*p* value ≤ ×10^−8^, fold change ≥ ±2). In total, 30,566 CpG sites were significantly different between the gDNA and clDNA samples within group 1 (n = 8 vs. n = 8, Additional file 2: Table S2), and 32,921 within group 2 (n = 8 vs. n = 8, Additional file 2: Table S3).

Furthermore, > 86% (28,632) of the CpG sites with significantly different levels of methylation were common to both groups. Only 6223 individual CpG sites differed in their level of methylation between groups 1 and 2, these are included in Additional file 2: Table S4. Therefore, 828,072 CpG sites assessed by this methylationEPIC array were not statistically different.

#### Differential methylation analysis between matched gDNA and clDNA samples for all samples

**Fig. 1:**
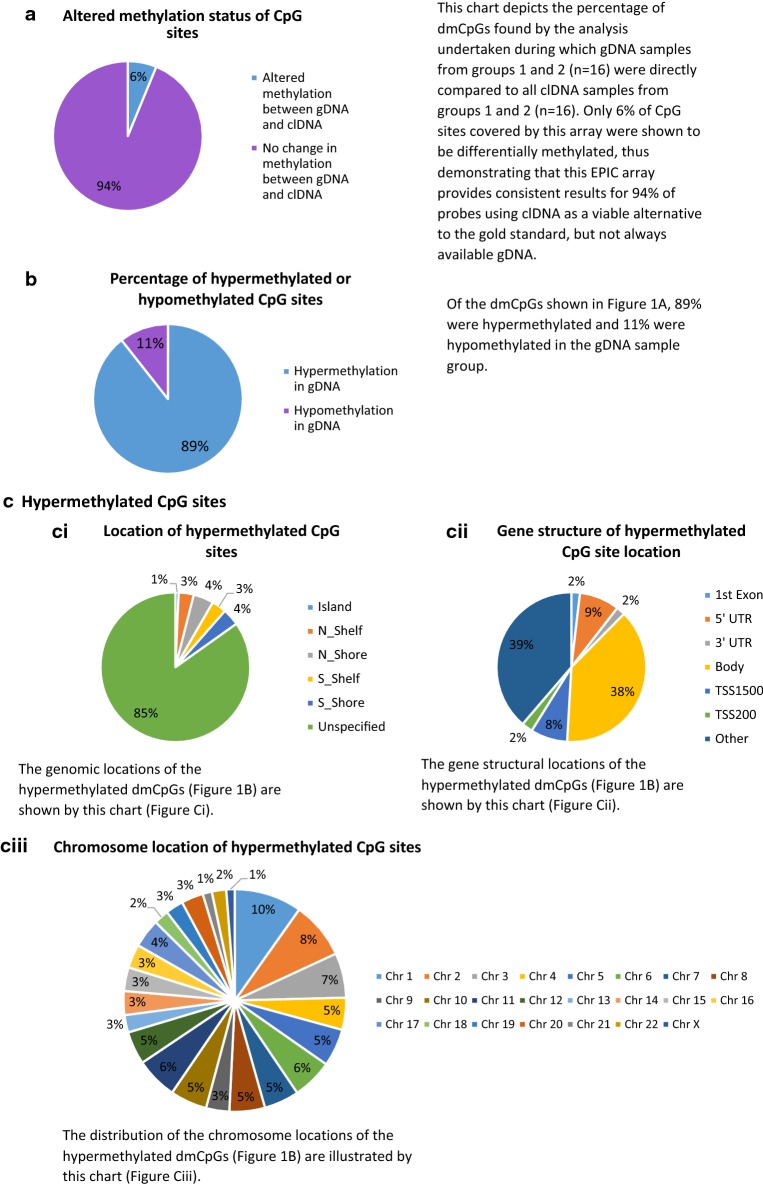

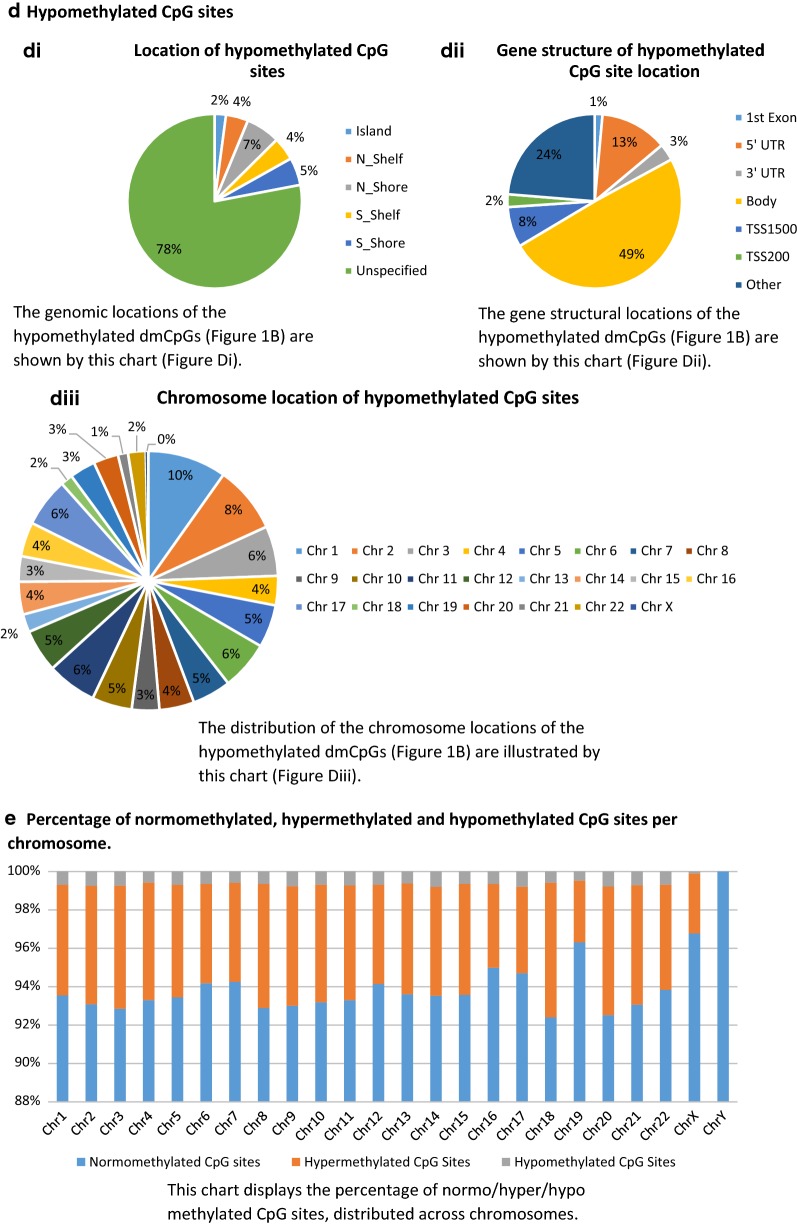
Comparison of differentially methylated CpG sites (dmCpGs) between gDNA and clDNA. **a** Altered methylation status of CpG sites between gDNA and clDNA; **b** Percentage of hypermethylated or hypomethylated CpG sites in gDNA; **c** Hypermethylated CpG sites; **d** Hypomethylated CpG sites; **e** Percentage of normomethylated, hypermethylated and hypomethylated CpG sites per chromosome. *CpG* cytosine-phosphate-guanine, *clDNA* cell-line DNA, *gDNA* genomic DNA, *N_Shelf* North Shelf, *N_Shore* North Shore, *S_Shelf* South Shelf, *S_Shore* South Shore, *TSS* transcription start site, *UTR* untranslated region

In the second analysis, all gDNA samples from groups 1 and 2 (n = 16) were directly compared to all clDNA samples from groups 1 and 2 (n = 16). These samples were matched (n = 16 vs. n = 16). Overall, 6.2% of the CpG sites covered by the array (53,764) were identified as having significantly different levels of methylation between the two groups (p ≤ ×10^−8^, fold change ≥ ±2). These results are included within and Additional file 2: Table S5 and an additional breakdown is available within Fig. 1.

In summary, of the 53,764 CpG sites which have shown differential methylation (1438/142,137 (1%) probe I; 52,326/720,790 (7.3%) probe II on this array), 89% were hypermethylated in the gDNA sample group. Of the hypermethylated CpG sites, 15% are located within islands (1%), shelves (6%) and shores (8%), and 38% within gene bodies. In comparison, 22% of the hypomethylated CpG sites were located within islands (2%), shelves (8%) and shores (12%) and 49% within gene bodies (Fig. 1). The chromosome location breakdown is similar for both hypermethylated and hypomethylated CpG sites. Additional file 3: Figure S1a–d are included to illustrate the pattern of average beta values, for each of the experimental groups.

#### GO and KEGG pathway analyses of differentially methylated genes

**Fig. 2:**
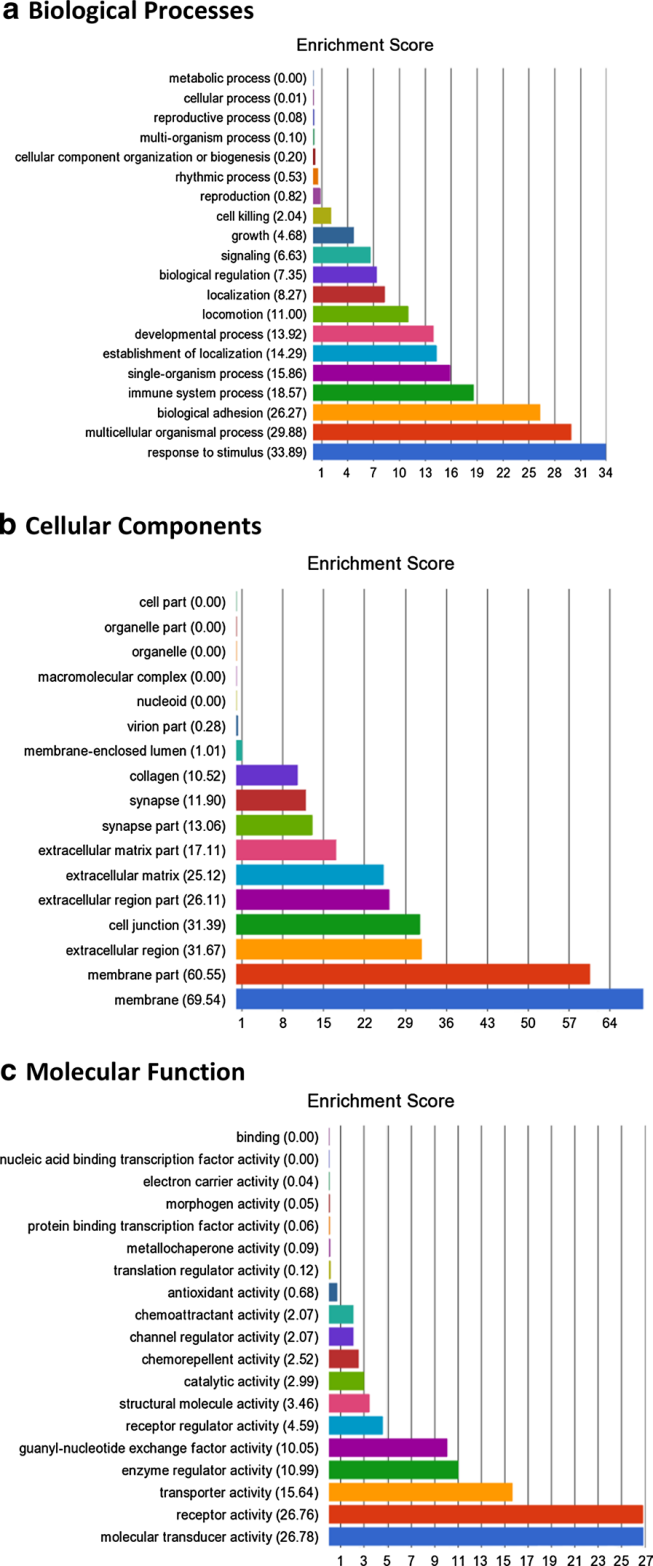
GO enrichment results. **a** Biological processes, **b** cellular components, **c** molecular functions. These results are determined from the enriched genes which house ≥ 1 top-ranked differentially methylated CpG site. The results refer to classes of genes in this population that are over represented and therefore may have an association with the disease phenotype compared to a control gene set

In order to assess the functional significance of the significant DNA methylation alterations between gDNA and clDNA, a GO enrichment analysis was undertaken. This assessed the biological processes, cellular components and molecular functions of the genes within which the top-ranked CpG sites were located. A total of 54 GO functions were found to have an enrichment score ≥ 10, alongside p ≤ ×10^−8^ and these are included within Additional file 2: Table S6 and Fig. 2. The processes with the top enrichment scores included signal transduction, signalling transduction activity, calcium ion binding, cell adhesion and immune system processes.

The KEGG pathway database was searched to identify key pathways linked to the genes where the top-ranked differentially methylated CpG sites were located. Eleven pathways were identified (an enrichment score of ≥ 8, and p ≤ ×10^−5^) which are included within Additional file 2: Table S7. This analysis has shown that differentially methylated genes are involved in pathways including focal adhesion, protein digestion and vascular and smooth muscle contraction.

#### Assessment of differential methylation between sample groups of the same origin

Lastly, the methylation status was quantitatively determined between the two sample types within groups 1 and 2; the gDNA (n = 8) samples in group 1 were directly compared to the gDNA samples (n = 8) in group 2. This was then repeated for the clDNA (n = 8 vs. n = 8) samples. Reassuringly, no CpG sites were significantly different between the two analysis groups.

### Discussion

This study reports a comparison of the data generated by Illumina’s Infinium MethylationEPIC BeadChip array technology for gDNA from peripheral blood leukocytes from 16 individuals and clDNA, derived from EBV transformation of the same samples into cell lines performed by the ECACC. The methylationEPIC BeadChip array covers 862,927 CpG sites, which makes this the largest gDNA and clDNA methylation profiling study using this array [15].

DNA methylation plays a key role in epigenetic gene regulation and is the most well studied epigenetic factor [16]. It has been shown to alter with age and smoking status and therefore it was important to align the two analysis groups for age of T1D diagnosis and duration of diabetes [17–19].

Through this analysis, we have established that approximately 6% of the CpG sites covered by the MethylationEPIC array provided significantly different p-values between gDNA and clDNA based on their methylation beta values. We have not addressed potential causes of the observed differences in methylation [20]. This may be due to the method through which the cell-line transformation occurs [21–23]. One study assessing oral keratinocytes [24] has shown that EBV infection itself affects methylation levels, resulting in alterations to gene expression. Consistent with Sugawara and colleagues, they also demonstrated that the epigenetic alterations were retained following removal of the virus [24, 25].

Furthermore, the cell-line passage number has been demonstrated to affect epigenetic modifications. Grafodatskaya et al. [26] compared methylation patterns in blood cells with lymphoblastoid cell-lines (LCLs) of different passage numbers. They showed that low passage numbers and one freeze–thaw cycle does not affect methylation, but identified that LCLs can be prone to alterations in the DNA methylation at sporadic genomic locations when at high passage numbers.

Two genes *AGXT* and *INS* which have CpG sites included within the 6223 CpG sites with a significant difference in methylation levels between gDNA vs. clDNA (groups 1 and 2), had previously also been shown to be differentially methylated in an investigation which assessed 25,000 CpG sites from six individuals by Brennan and colleagues between gDNA and clDNA [21].

We have also shown that the CpG sites with differential methylation were not due to differences between the individuals within the two groups. Neither comparison, gDNA (group 1 vs. group 2), nor clDNA (group 1 vs. group 2) provided any significantly different levels of methylation.

We have previously shown that clDNA is a suitable replacement for gDNA in SNP-based analyses [27] and it is evident from these results, that the clDNA has potential to be an alternative material source for assessment of DNA methylation using the methylationEPIC array.

## Limitations

A potential limitation is that the methylation data was generated for only 16 matched gDNA and clDNA samples, compared in groups of eight samples. It would be advantageous to repeat this on a larger sample size. As the cell line DNA was prepared off-site, it is possible that the number of freeze–thaw cycles of the two different collections could have been different, but we believe there were less than three freeze–thaw cycles for each aliquot.

## Supplementary information

**Additional file 1: Appendix S1.** Illustration of methods.

**Additional file 2: Table S1.** Illumina Bead Array Controls Reporter for the gDNA and clDNA samples. **Table S2.** Top-ranked differentially methylated CpG sites for gDNA vs. clDNA (group 1). **Table S3.** Top-ranked differentially methylated CpG sites for gDNA vs. clDNA (group 2). **Table S4.** Top-ranked CpG sites which differed between groups 1 and 2 (gDNA vs. clDNA - ST2 and ST3). **Table S5.** Top-ranked CpG sites which were common between groups 1 and 2 (gDNA vs. clDNA - ST2 and ST3). **Table S6.** GO enrichment analysis for genes where top-ranked CpG sites are located. **Table S7.** Enriched pathways (KEGG) for genes where top-ranked CpG sites are located.

**Additional file 3: Figure S1.** Illustration of the average methylation beta value patterns generated for each of the four groups. A) Group 1 (gDNA); B) Group 1 (clDNA); C) Group 2 (gDNA); D) Group 2 (clDNA).

## Data Availability

The datasets generated and/or analysed during the current study are available from the corresponding author on reasonable request.

## Abbreviations

BMI: body mass index
BP: blood pressure
BST: bisulphite treated
CKD: chronic kidney disease
clDNA: cell-line derived DNA
CpG: cytosine-phosphate-guanine
DCs: diabetic controls
DKD: diabetic kidney disease
DNA: deoxyribose nucleic acid
EBV: Epstein–Barr virus
ECACC: European Collection of Authenticated Cell Cultures
eGFR: estimated glomerular filtration rate
ESRD: end-stage renal disease
gDNA: genomic DNA
GO: gene ontology
GoKinD: Genetics of Kidneys in Diabetes
KEGG: Kyoto Encyclopedia of Genes and Genomes
LCLs: lymphoblastoid cell-lines
mmHg: millimetres of mercury
QC: quality control
T1D: type 1 diabetes
T1DKD: type 1 diabetes and kidney disease
WGBS: whole-genome bisulphite sequencing
