# Supplementary material for: Comparison of methylation patterns generated from genomic and cell-line derived DNA using the Illumina Infinium MethylationEPIC BeadChip array
Source: BMC Res Notes. 2019 Dec 21;12:821. doi: 10.1186/s13104-019-4853-4 (PMC6925854; doi:10.1186/s13104-019-4853-4)

Appendix S1: Illustration of methods

- There are two groups in this analysis: group 1 (discovery group) and group 2 (replication group)
- Both groups contain 16 DNA samples, from 8 individuals, providing 8 genomic DNA and 8 cell line DNA samples. There is a gDNA and a clDNA sample per participant
- Participants with T1DKD are represented by a blue circle, those with T1D and no evidence of kidney disease are represented by a green circle – this disease phenotype information is only to ensure both groups are matched for age, sex, and phenotype
- In the diagram below, the four participants with T1DKD in group 1 are matched to the four participants with T1DKD in group 2
- The same setup is applied for the four participants with T1D only

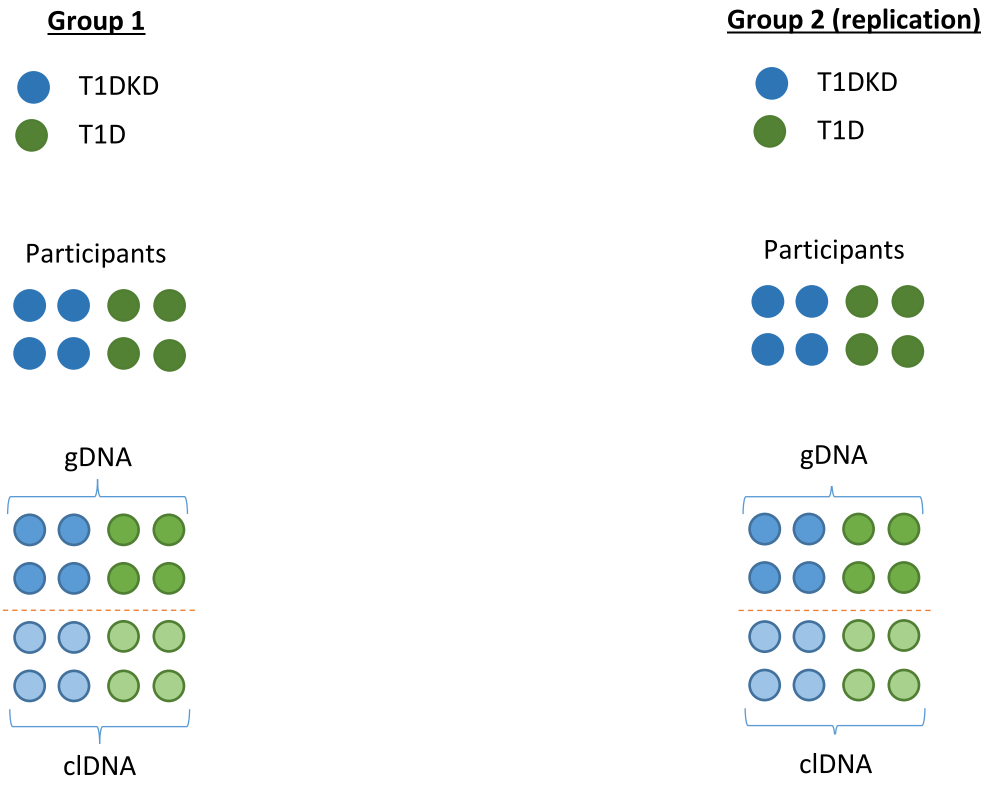

- ***Analysis 1a:*** Assess differentially methylated CpG sites (dmCpGs) between gDNA and clDNA for group 1. The disease phenotypes are not under investigation.
    - Compare the methylation signatures for all 8 gDNA samples to all 8 clDNA samples
    - Number of differentially methylated CpG sites determined

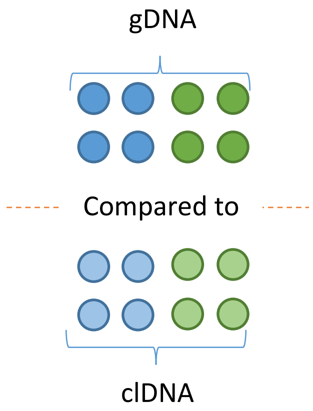

- ***Analysis 1b:*** Assess dmCpGs between gDNA and clDNA for group 2
    - Compare the methylation signatures for all 8 gDNA samples to all 8 clDNA samples
    - Number of differentially methylated CpG sites determined

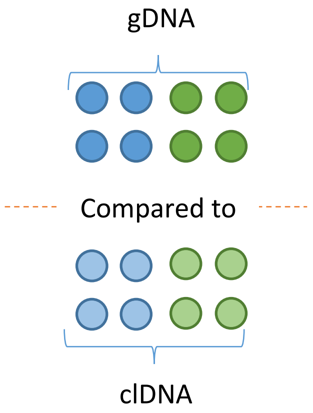

- ***Analysis 1c:*** Assess overlapping dmCpGs between gDNA and clDNA
- ***Analysis 1d:*** Determine the CpG sites unique to the comparison of groups 1 and 2

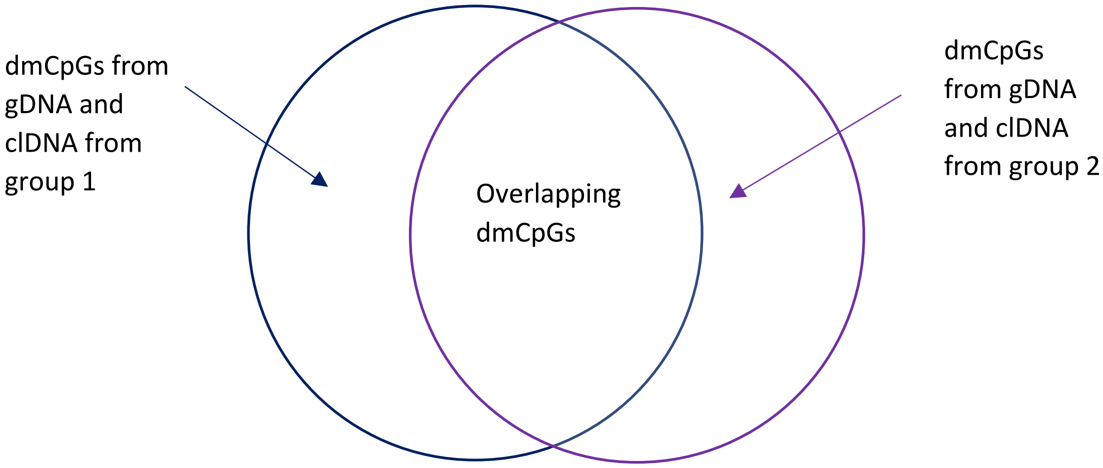

- **Analysis 2:** Assess dmCpGs between gDNA and clDNA for all samples. The disease phenotypes are not under investigation.

    - The methylation status was quantitatively determined between the two sample types
    - The gDNA (n=16) samples originally from groups 1 and 2 were directly compared to the equivalent clDNA samples (n=16), again from groups 1 and 2

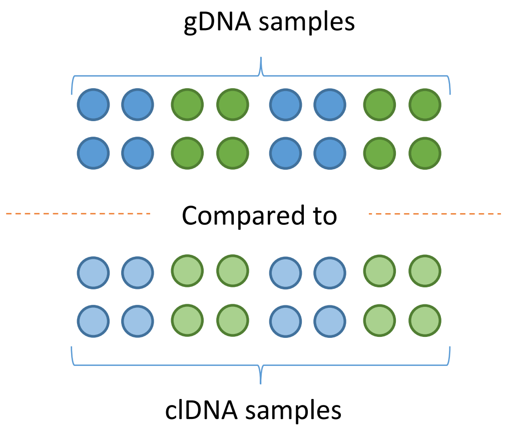

- **Analysis 3:** Assessment of differential methylation between sample groups of the same origin
    - The methylation status was quantitatively determined between the two sample types within groups 1 and 2
    - The gDNA (n=8) samples from group 1 were directly compared to the gDNA samples (n=8) from group 2
    - This was then repeated for the clDNA (n=8 vs. n=8) samples

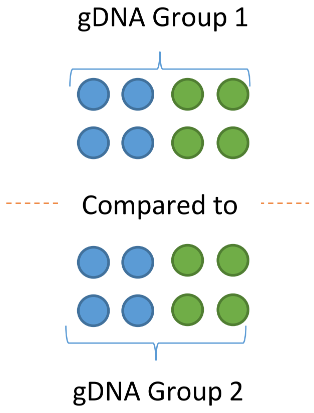

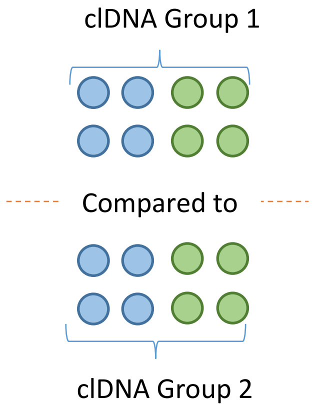

Supplement: Supplementary file 1 — Additional file 1: Appendix S1. Illustration of methods. [file 13104_2019_4853_MOESM1_ESM.pdf]
