# Supplementary material for: Comparison of methylation patterns generated from genomic and cell-line derived DNA using the Illumina Infinium MethylationEPIC BeadChip array
Source: BMC Res Notes. 2019 Dec 21;12:821. doi: 10.1186/s13104-019-4853-4 (PMC6925854; doi:10.1186/s13104-019-4853-4)

**Figures S1:** Illustration of the average methylation beta value patterns generated for each of the four groups. A) Group 1 (gDNA); B) Group 1 (clDNA); C) Group 2 (gDNA); D) Group 2 (clDNA).

**A)**


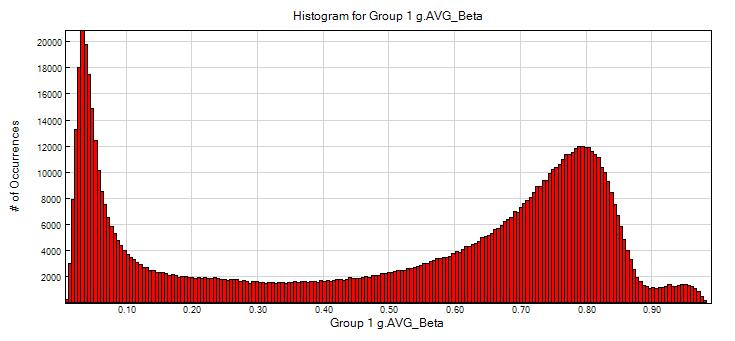


**B)**


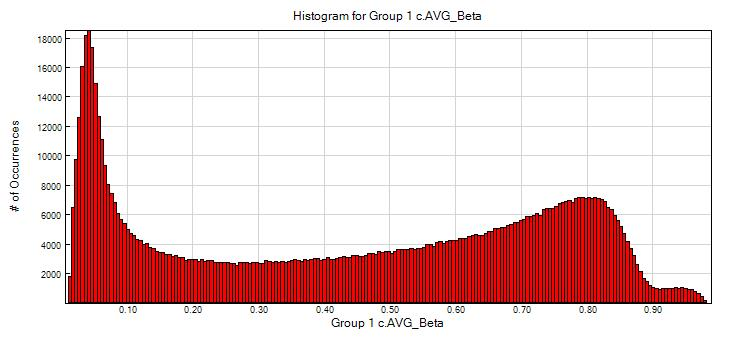


**C)**


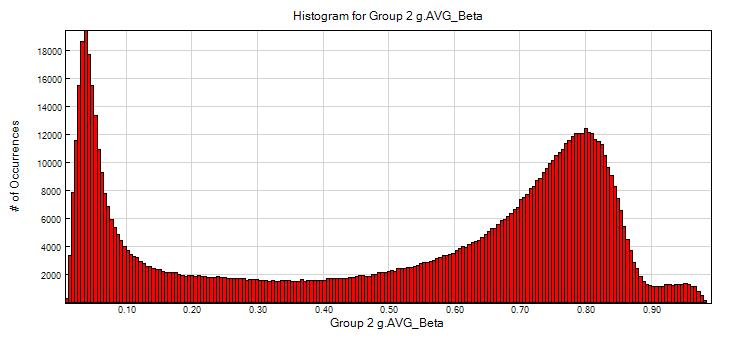


**D)**


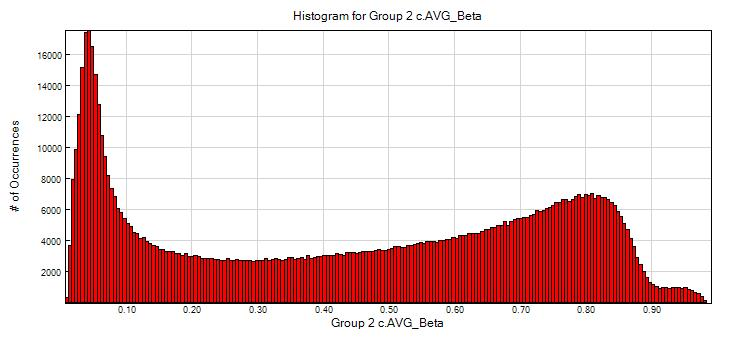

Supplement: Supplementary file 3 — Additional file 3: Figure S1. Illustration of the average methylation beta value patterns generated for each of the four groups. A) Group 1 (gDNA); B) Group 1 (clDNA); C) Group 2 (gDNA); D) Group 2 (clDNA). [file 13104_2019_4853_MOESM3_ESM.docx]
